# Structure and Electrochemical Properties of Mn_3_O_4_ Nanocrystal-Coated Porous Carbon Microfiber Derived from Cotton

**DOI:** 10.3390/ma11101987

**Published:** 2018-10-15

**Authors:** Dongya Sun, Liwen He, Yongle Lai, Jiqiong Lian, Jingjing Sun, An Xie, Bizhou Lin

**Affiliations:** 1 Key Laboratory of Functional Materials and Applications of Fujian Province, School of Materials Science and Engineering, Xiamen University of Technology, Xiamen 361024, China; heliwen@hqu.edu.cn (L.H.); 2013123201@xmut.edu.cn (Y.L.); 2013123202@xmut.edu.cn (J.L.); 2015000093@xmut.edu.cn (J.S.); anxie@xmut.edu.cn (A.X.); 2 Fujian Key Laboratory of Photoelectric Functional Materials, College of Materials Science and Engineering, Huaqiao University, Xiamen 361021, China

**Keywords:** Mn_3_O_4_, carbon microfibers, biotemplate, microstructure, energy storage and conversion, electrochemical properties

## Abstract

Biomorphic Mn_3_O_4_ nanocrystal/porous carbon microfiber composites were hydrothermally fabricated and subsequently calcined using cotton as a biotemplate. The as-prepared material exhibited a specific capacitance of 140.8 F·g^−1^ at 0.25 A·g^−1^ and an excellent cycle stability with a capacitance retention of 90.34% after 5000 cycles at 1 A·g^−1^. These characteristics were attributed to the introduction of carbon fiber, the high specific surface area, and the optimized microstructure inherited from the biomaterial.

## 1. Introduction

Electrochemical supercapacitors (ESs) have many desirable properties, including long lifetime, high power density, and high rate capability. Thus, ESs are attracting worldwide attention as an efficient energy storage device for portable electronic devices and vehicles [1,2]. Transition metal oxides (TMOs) such as CoO_*x*_, NiO, and MnO_*x*_ have been extensively investigated as promising electrode materials for ES applications. These oxides deliver higher specific capacitances than those of carbonaceous materials due to reversible faradaic redox reactions [2]. Manganese oxide has been considered a highly attractive TMO due to its high theoretical specific capacitance, good electrochemical stability, low cost, and natural abundance [3]. MnO_*x*_-based composites with various microstructures and morphologies, such as wires, sheets, tubes, and flowers, have been developed [4,5,6,7]. Although these active materials exhibit enhanced pseudocapacitance properties, their low electronic conductivity and insufficient interface contact can substantially reduce the experimental specific capacitance and hamper their extensive commercial application.

To address these issues, scholars have proposed the fabrication of many MnO_*x*_/carbon composites in recent years [8,9]. Among the carbon materials, using low-cost natural resources, biowaste, and food waste are highly effective ways to achieve the large-scale production of electrode materials [10,11,12]. Natural biomaterials usually possess irregular microstructures that are difficult to duplicate, and these structures are often highly suitable as energy storage materials. For example, cotton wool [6], coconut shell [13], and human hair [14] have been introduced as excellent flexible carbon substrates for hybrid composites. These materials exhibit high specific capacitance, good electronic conductivity, good cycle stability, and excellent flexibility. Therefore, a simple and cheap approach using porous carbon microfiber (PCM) derived from cotton wool was studied in our work.

Herein, we prepared a biomorphic porous composite fabricated using Mn_3_O_4_ nanocrystals supported on PCMs (Mn_3_O_4_/PCM) with cotton fiber as the biotemplate and carbon skeleton. Benefitting from the unique morphology, large specific surface area, and existence of carbon fiber, the as-prepared composites exhibit an excellent electrochemical performance.

## 2. Experimental

**Scheme 1:**
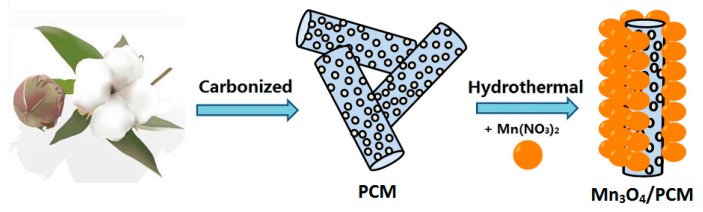
Illustration of the preparation process of Mn_3_O_4_/PCM (porous carbon microfiber).

The preparation process of Mn_3_O_4_/PCM is schematically illustrated in Scheme 1. Dry cotton wool (Xinghua health cotton wool Co. LTD, Xinghua, China) was cut into short pieces and carbonized for 1 h at 350 °C under an argon atmosphere. The as-prepared sample is denoted as PCM. Then, 2.5 g PCM was immersed in 50 mL of 1 mol·L^−1^ Mn(NO_3_)_2_·6H_2_O solution. The dispersed solution was transferred to a 100 mL autoclave and reacted for 12 h at 180 °C. The hydrothermal reaction product was filtered, washed, dried, and annealed in a tubular furnace at 400 °C in air for 1 h, and the product was labeled as Mn_3_O_4_/PCM. For comparison, pristine Mn_3_O_4_ was hydrothermally synthesized by decomposing the solution of Mn(NO_3_)_2_·6H_2_O at 180 °C for 12 h without cotton.

Field emission scanning electron microscopy (FE-SEM) images were taken with a Zeiss Sigma 500 microscope (Carl Zeiss A G, Jena, German) and an Oxford X-Max energy dispersive spectroscope (EDS, Oxford instruments, Oxford, UK). Transmission electron microscopy (TEM) images were taken using a FEI Talos F200S microscope (Thermo fisher Scientific, Waltham, MA, USA) with an accelerating voltage of 200 kV. Powder X-ray diffraction (XRD) patterns were obtained at ambient temperature on a Rigaku Smart Lab 3 kW diffractometer (Rigaku, Tokyo, Japan) using Cu K radiation (λ = 1.5418 Å) under an accelerating voltage of 36 kV. Raman spectroscopy was tested with the range of 300–2000 cm^−1^ on a LABRAM HR-800 spectrometer (Horiba, Kyoto, Japan), and the excitation source was a 532 nm laser. Specific surface area and porosity measurements were carried out on a Quantachrome Autosorb-iQ instrument (Quantachrome instruments, Boynton Beach, FL, USA) using the Brunauer–Emmett–Teller (BET) method. X-ray photoelectron spectroscopy (XPS) measurements were performed on a VG Escalab MK II spectrometer (Thermo fisher Scientific, Waltham, MA, USA) with non-monochromatic Al Kα X-ray (1486.6 eV). The TG-DTA were measured on an integrated thermal analyzer (TG, STA 449C, NETZSCH-Gerätebau GmbH, Selb, Germany) with a 10 °C/min heating rate from room temperature to 750 °C under air atmosphere. The working electrode was prepared by mixing the prepared composites, acetylene black, and polytetrafluoroethylene with a mass ratio of 75:15:10, while electrode of the bulk Mn_3_O_4_ was 50:40:10. The net weight of Mn_3_O_4_ in electrodes was ~3 mg. Subsequently, the mixture was coated onto a nickel foam current collector (1.5 cm^2^), pressed at 10 MPa, and dried under vacuum at 60 °C. All the measurements were performed with a three-electrode system (Hg/HgO and platinum as the reference and counter electrode, respectively) and a two-electrode system (Mn_3_O_4_/PCM and commercial active carbon (CAC)) in 3 mol·L^−1^ KOH aqueous electrolyte.

## 3. Results and Discussion

**Figure 1:**
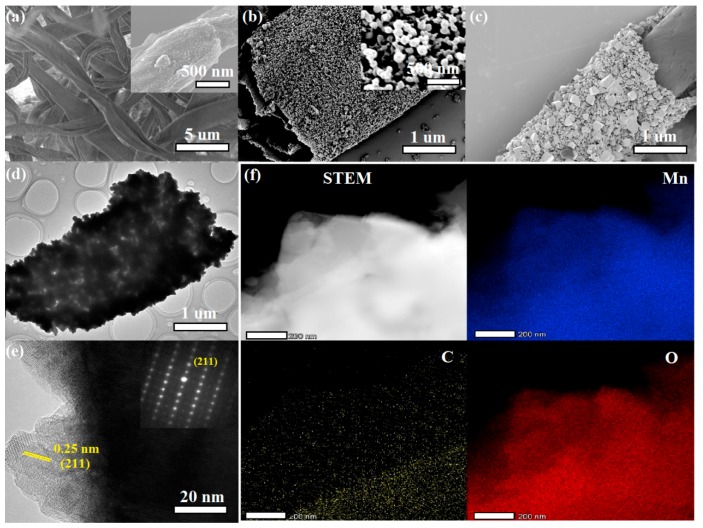
SEM images of (**a**) cotton and carbonized cotton fiber (inset in a); (**b**,**c**) Mn_3_O_4_ nanocrystals grown on the PCM surface after 5 and 12 h, respectively. (**d**) Transmission electron microscopy (TEM) image, (**e**) high-resolution TEM (HRTEM) image (inset is the SEAD graph), and (**f**) energy dispersive spectroscopy (EDS) mappings of Mn_3_O_4_/PCM.

The carbon fiber of the cotton became remarkably porous after carbonization (Figure 1a). The Mn_3_O_4_/PCM composite prepared using cotton as template had a tubular morphology (Figure 1b,c) similar to cotton. At the start of the hydrothermal reaction, the cotton was immersed into the precursor solution, the Mn^2+^ was partly oxidized to Mn^3+^ ions, and Mn_3_O_4_ formed. Mn ions were then bonded onto the oxygen-rich group of the cotton fibers through O‒H, C=O groups, and so on (Figure 1b) [15,16,17]. The size of nanocrystals increased from several to dozens of nanometers (Figure 1b,c) with a reaction time from 5 h to 12 h. When the hydrothermal product was calcined at 400 °C, the precise replication of cotton texture could be achieved after the removal of most of the template substance. Part of the organic template was carbonized into a porous carbon stick with irregular pores (Figure 1d). The high-resolution TEM (HRTEM) image (Figure 1e) clearly demonstrates that the biomorphic Mn_3_O_4_ was monocrystalline and derived from the lattice arrangement (inset of Figure 1e). The interplanar spacing was about 0.25 nm for the {211} lattice planes. The distribution of Mn (44.8 wt.%), C (31.7 wt.%), and O (23.5 wt.%) in the as-prepared sample was uniform (Figure 1f).

**Figure 2:**
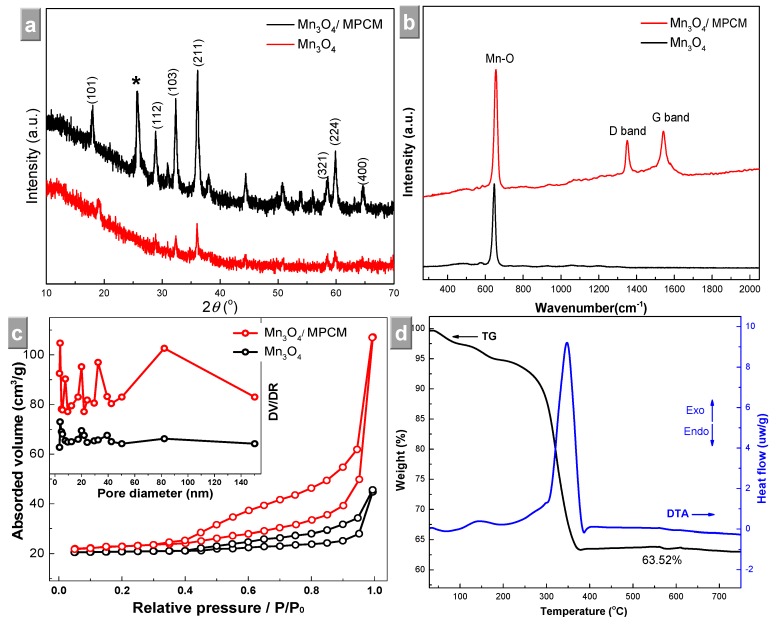
(**a**) XRD patterns, (**b**) Raman spectra, (**c**) nitrogen adsorption–desorption isotherms (inset shows pore size distributions) of Mn_3_O_4_/PCM and bulk Mn_3_O_4_, and (**d**) TG-DTA curves of the product.

The XRD patterns of Mn_3_O_4_/PCM and bulk Mn_3_O_4_ are shown in Figure 2a. The diffraction peaks of the composite can be ascribed to tetragonal Mn_3_O_4_ (JCPDS 18-0803) [18], and the diffraction peak at 25.24° was ascribed to the carbon fabric (graphitic carbon: JCPDS75-1621), which was marked with an asterisk symbol (*). Raman characterizations (Figure 2b) showed features from Mn–O in 600–700 cm^−1^ and carbon fibers in the 1200‒1600 cm^−1^ region in Mn_3_O_4_/PCM [4,9,16], while only an Mn–O peak at lower wavenumber in Mn_3_O_4_.

The N_2_ adsorption–desorption isotherm of Mn_3_O_4_/PCM exhibited a distinct H3 hysteresis loop at *p*·*p*_0_^−1^ > 0.40 (Figure 2c). The isotherms of composite and pristine Mn_3_O_4_ exhibited features of mesoporous materials. The pore size distribution of Mn_3_O_4_/PCM appeared with a wide range having two pore extremes at 24.2 and 82.5 nm. The BET specific surface area of the Mn_3_O_4_/PCM was determined to be 51.9 m^2^·g^−1^, which was much larger than that of pristine Mn_3_O_4_ (10.8 m^2^·g^−1^). The higher surface area and pore volume is beneficial to the contact of the electrolytes with the active materials, and can further increase the electrochemical properties [6,8,10]. The TG-DTA curves of the hydrothermal product are shown in Figure 2d. Two endothermic peaks were observed in the range of 100‒200 °C because of the adsorbed water. A weight loss of ~30% was found between 250 and 380 °C, which can be attributed to the decomposition of organic substances in cotton similar to other biomorphic oxides [13,14].

**Figure 3:**
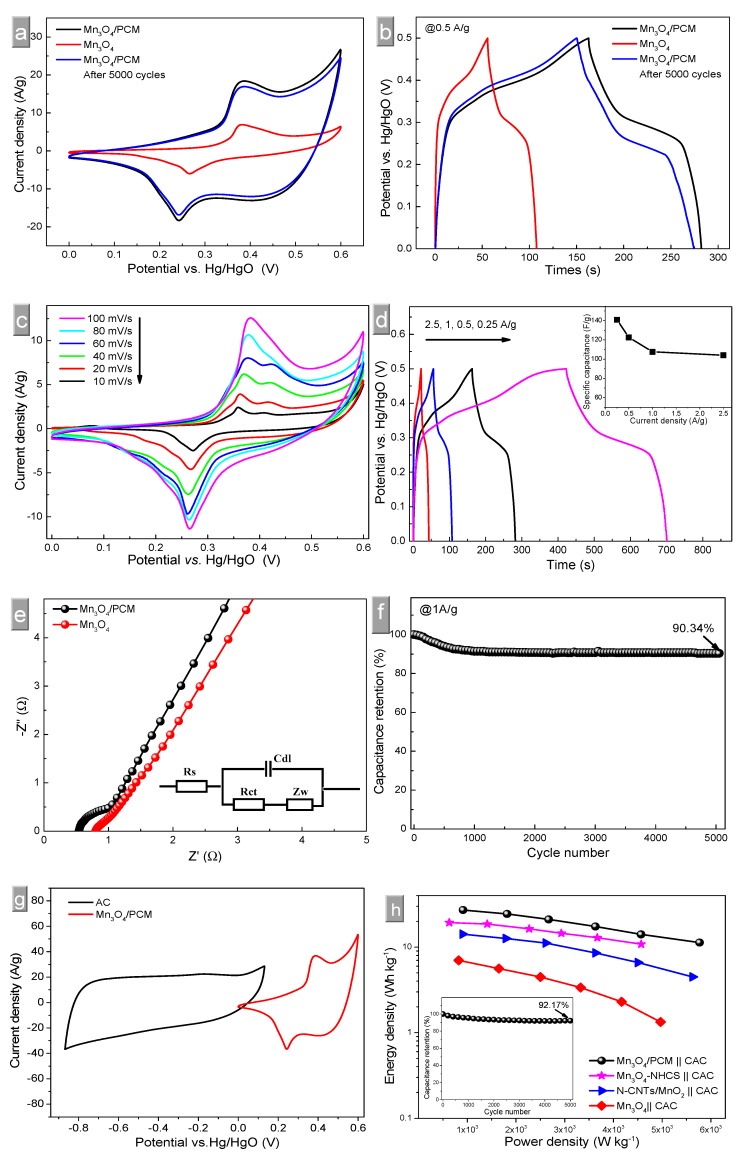
(**a**) Cyclic voltammetry (CV) curves at a scan rate of 80 mV·s^−^^1^; (**b**) galvanostatic charge–discharge (GCD) curves at 0.5 A·g^−^^1^ of Mn_3_O_4_/PCM and bare Mn_3_O_4_; (**c**) CV curves of Mn_3_O_4_/PCM at various scan rates; (**d**) GCD curves and specific capacitance at various current densities of Mn_3_O_4_/PCM (inset in d); (**e**) Nyquist plots of Mn_3_O_4_/PCM and bare Mn_3_O_4_; (**f**) capacitance retention of Mn_3_O_4_/PCM vs. cycle number at 1 A·g^−^^1^; (**g**) CV curves of Mn_3_O_4_/PCM and CAC at 20 mV·s^−^^1^; and (**h**) Ragone plots of asymmetrical supercapacitor in MnO_*x*_-based two-electrodes systems. CAC: commercial active carbon.

As shown in Figure 3a,b, the cyclic voltammetry (CV) and galvanostatic charge–discharge (GCD) curves of Mn_3_O_4_/PCM and bare Mn_3_O_4_ demonstrated that the electrochemical properties of the former were better than those of the latter. The specific capacitances were calculated to be 120.8 and 52.2 F·g^−^^1^, respectively, which were attributed to the higher specific surface area of Mn_3_O_4_/PCM, the existence of carbon fiber, and its special hierarchical porous morphology derived from the biotemplate. As illustrated in Figure 3c, Mn_3_O_4_/PCM and pristine Mn_3_O_4_ had a pair of redox peaks located at ~0.25 and ~0.4 V, which correspond to almost the same component and chemical conversion between different manganese oxidation states in alkaline medium (Mn_3_O_4_ ⇔ MnOOH) [18,19]. The GCD curves of Mn_3_O_4_/PCM displayed remarkable pseudocapacitance properties. A slight reduction of specific capacitance was noted at high current densities (Figure 3d), indicating that the as-prepared composite has a good electrochemical stability [4,14].

The electrochemical impedance spectroscopy (EIS) measurements of the biomorphic Mn_3_O_4_/PCM and bulk Mn_3_O_4_ are shown in Figure 3e. The charge-transfer resistance (*R*_ct_) (radius of the semicircle) of biomorphic electrode (≤0.5 Ω) was obviously lower than that of pristine Mn_3_O_4_ (>0.6 Ω), suggesting the substantially improved reaction kinetics (e.g., increased charge transfer rate) for microtubular Mn_3_O_4_/PCM. The increase in rate enables additional rapid redox reaction and facilitates electron transport, and thus improves the specific capacitance. As shown in Figure 3f, the long-term cycling stability of Mn_3_O_4_/PCM electrode was tested at 1 A·g^−1^ for 5000 cycles. This result indicates that the electrode maintained more than 90% of its initial value after 5000 cycles, suggesting the excellent cyclic stability of the electrode. At the same time, the CV and GCD profiles after 5000 cycles migrated slightly from values before cycling (Figure 3a,b). The superior electrochemical performance of Mn_3_O_4_/PCM can be attributed to the special tubular morphology, high specific surface area, and porous carbon microfibers inside.

After assembling into an asymmetric supercapacitor using the two-electrode system of Mn_3_O_4_/PCM and CAC, the working voltage could be expanded to 0–1.4 V (Figure 3g) while the three-electrode system was 0–0.6 V. As shown in Figure 3h, the specific capacitance of Mn_3_O_4_/PCM||CAC was 110.8 F·g^−1^ at a current density of 1 A·g^−1^. The inset of Figure 3h is a Ragone plot of the asymmetric electrode. The energy density of the cell configuration was 27.13 Wh·kg^−1^ at a power density of 0.41 kW·kg^−1^. Even at a high power density of 4.76 kW·kg^−1^, the energy density still maintained at 11.34 Wh·kg^−1^. Our results were better than previous reports concerned with special morphologies of Mn_3_O_4_-based composites used as supercapacity electrodes (Figure 3h) [4,20]. There was still specific capacitance of 92.17% retained in the cell after 5000 cycles (inset of Figure 3h), suggesting that the electrode has a high reversibility and good electrochemical stability.

## 4. Conclusions

This work successfully prepared a biomorphic Mn_3_O_4_/PCM composite via a simple hydrothermal route by using cotton as a biotemplate. The as-obtained Mn_3_O_4_/PCM was composed of Mn_3_O_4_ nanocrystal-coated carbon fiber, inherited the morphology and microstructure of cotton, and exhibited the excellent electrochemical properties of a specific capacitance of 140.8 F·g^−^^1^ at 0.25 A·g^−^^1^ and long cycling life with 90.34% of the capacitance after 5000 cycles. This work provides an example of the fabrication of biomorphic porous TMOs for developing promising electrode materials in energy storage and conversion.

## References

[1] Zhang Y.Z., Wang Y., Cheng T., Lai W.Y., Pang H., Huang W. (2015). Flexible supercapacitors based on paper substrates: A new paradigm for low-cost energy storage. Chem. Soc. Rev..

[2] Li B., Dai F., Xiao Q., Yang L., Shen J., Zhang C., Cai M. (2016). Nitrogen-doped activated carbon for high energy hybrid supercapacitor. Energy Environ. Sci..

[3] Huang M., Li F., Dong F., Zhang Y.X., Zhang L.L. (2015). MnO_2_-based nanostructures for high-performance supercapacitors. J. Mater. Chem. A.

[4] Liu T., Jiang C., You W., Yu J. (2017). Hierarchical porous C/MnO_2_ composite hollow microspheres with enhanced supercapacitor performance. J. Mater. Chem. A.

[5] Zang J., Ye J.C., Qian H., Lin Y., Zhang X.W., Zheng M.S., Dong Q.F. (2018). Hollow carbon sphere with open pore encapsulated MnO_2_ nanosheets as high-performance anode materials for lithium ion batteries. Electrochim. Acta.

[6] Yan D.L., Li S.C., Zhu G.S., Wang Z.M., Xu H.R., Yu A.B. (2013). Synthesis and pseudocapacitive behaviors of biomorphic mesoporous tubular MnO_2_ templated from cotton. Mater. Lett..

[7] Zhou Y., Guo L., Shi W., Zou X.F., Xiang B., Xing S.H. (2018). Rapid production of Mn_3_O_4_/rGO as an efficient electrode material for supercapacitor by flame plasma. Materials.

[8] Aveiro L.R., Silva A.G.M.D., Antonin V.S., Candido E.G., Parreira L.S., Geonmonond R.S., Freitas I.C.D., Lanza M.R.V., Camargo P.H.C., Santos M.C. (2018). Carbon-supported MnO_2_ nanoflowers: Introducing oxygen vacancies for optimized volcano-type electrocatalytic activities towards H_2_O_2_ generation. Electrochim. Acta.

[9] Yan D.L., Zhang H., Chen L., Zhu G.S., Li S.C., Xu H.R., Yu A.B. (2014). Biomorphic synthesis of mesoporous Co_3_O_4_ microtubules and their pseudocapacitive performance. ACS Appl. Mater. Interfaces.

[10] Wang J., Nie P., Ding B., Dong S., Hao X., Dou H., Zhang X. (2016). Biomass derived carbon for energy storage devices. J. Mater. Chem. A.

[11] Liu Y., Lv B., Liu P., Chen Y., Gao B., Lin B. (2018). Biotemplate-assisted hydrothermal synthesis of tubular porous Co_3_O_4_ with excellent charge-discharge cycle stability for supercapacitive electrodes. Mater. Lett..

[12] Cakici M., Kakarla R.R., Alonso-Marroquin F. (2017). Advanced electrochemical energy storage supercapacitors based on the flexible carbon fiber fabric-coated with uniform coral-like MnO_2_ structured electrodes. Chem. Eng. J..

[13] Sun L., Tian C.G., Li M.T., Meng X.Y., Wang L., Wang R.H., Yin J., Fu H.G. (2013). From coconut shell to porous graphene-like nanosheets for high-power supercapacitors. J. Mater. Chem. A.

[14] Saravanan K.R., Kalaiselvi N. (2015). Nitrogen containing bio-carbon as a potential anode for lithium batteries. Carbon.

[15] Tong G., Liu Y., Guan J. (2014). In situ gas bubble-assisted one-step synthesis of polymorphic Co_3_O_4_, nanostructures with improved electrochemical performance for lithium ion batteries. J. Alloys Compd..

[16] Sun D.Y., He L.W., Chen R.Q., Lin Z.Y., Lin S.S., Xiao C.X., Lin B.Z. (2018). The synthesis, characterization and electrochemical performance of hollow sandwich microtubules composed of ultrathin Co_3_O_4_ nanosheets and porous carbon by bio-template. J. Mater. Chem. A.

[17] Sun D.Y., He L.W., Chen R.Q., Liu Y., Lv B.J., Lin S.S., Lin B.Z. (2019). Biomorphic composites composed of octahedral Co_3_O_4_ nanocrystals and mesoporous carbon microtubes templated from cotton for excellent supercapacitor electrodes. Appl. Surf. Sci..

[18] Wang L., Duan G.R., Chen S.M., Liu X.H. (2018). Hydrothermally controlled synthesis of α-MnO_2_, γ-MnOOH, and Mn_3_O_4_ nanomaterials with enhanced electrochemical properties. J. Alloys Compd..

[19] Zhou J., Zhao H., Mu X., Chen J., Zhang P., Wang Y., He Y., Zhang Z., Pan X., Xie E. (2015). Importance of polypyrrole in constructing 3D hierarchical carbon nanotube@MnO_2_ perfect core-shell nanostructures for high-performance flexible supercapacitors. Nanoscale.

[20] Zhu J., Xu Y., Hu J., Wei L., Liu J., Zheng M. (2018). Facile synthesis of MnO_2_ grown on nitrogen-doped carbon nanotubes for asymmetric supercapacitors with enhanced electrochemical performance. J. Power Sources.

